# A Case Report on a Bilateral Simultaneous Olecranon Apophyseal Fracture After a Simple Fall: Initial Diagnosis of Osteogenesis Imperfecta

**DOI:** 10.7759/cureus.79883

**Published:** 2025-03-01

**Authors:** Nezih Ziroglu, Emre Tunçcan, Gulsah Ziroglu, Yasemin Şahbaz

**Affiliations:** 1 Orthopedics and Traumatology, Acibadem University Hospital Atakent, Istanbul, TUR; 2 Pediatrics, Arnavutkoy Public Hospital, Istanbul, TUR; 3 Physical Therapy, Istanbul Beykent University, Istanbul, TUR

**Keywords:** bisphosphonate use, brittle bone, elbow trauma, olecranon fractures, osteogenesis imperfecta

## Abstract

Osteogenesis imperfecta (OI) is a hereditary disorder characterized by abnormal type I collagen metabolism, leading to increased bone fragility. Fractures, often occurring with minor trauma, may manifest in atypical patterns in affected children. Among these, olecranon fractures (OFs) pose a particular risk in pediatric patients with OI. We present the case of a 12-year-old boy with undiagnosed OI type 1 who sustained bilateral simultaneous olecranon avulsion fractures following a simple fall. To our knowledge, this case is the first reported instance in the literature of simultaneous bilateral apophyseal OF in a patient with previously undiagnosed OI. We aim to highlight the importance of considering OI in the differential diagnosis of pediatric OF encountered by orthopedic surgeons. This case underscores the need for heightened clinical suspicion, emphasizing the significance of a holistic approach to patients and detailed history-taking for early diagnosis and appropriate management in children with OI to optimize outcomes.

## Introduction

Osteogenesis imperfecta (OI) is a genetic disorder of connective tissue characterized by increased bone fragility and low bone mass due to mutations in the genes encoding type I collagen, particularly COL1A1 and COL1A2 [1]. Patients with OI often present with a history of fractures from atraumatic or low-energy trauma, joint hypermobility, short stature, blue-gray sclera, hearing loss, and dentinogenesis imperfecta [2].

While olecranon fractures (OFs), occurring at the bony prominence of the elbow, are rare in children, they are more common in those with OI compared to the general pediatric population [3]. In children with OI, purely apophyseal fractures have been described more frequently. They are characterized by an indirect mechanism of injury and an avulsion fracture, wherein the olecranon tip becomes detached due to a sudden contraction of the triceps brachii muscle [4,5].

This case describes a 12-year-old boy with OI who sustained bilateral simultaneous olecranon apophyseal fractures following a simple fall. This case underscores the importance of considering OI as a potential diagnosis in pediatric patients with OF. By raising awareness, we aim to ensure that OI is included in the differential diagnosis portfolio of orthopedic surgeons to prevent further declines in quality of life and more severe outcomes. To our knowledge, this is the first reported OI case diagnosed after a bilateral simultaneous OF.

## Case presentation

**Figure 1:**
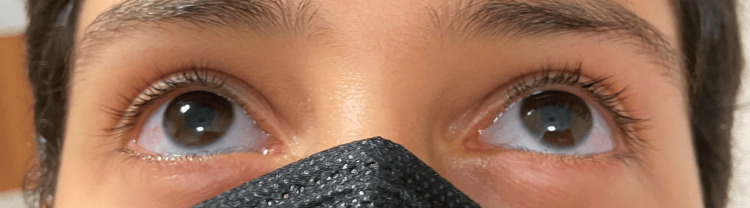
Blue sclerae, characteristic of osteogenesis imperfecta type 1.

A 12-year-old boy, 161 cm tall and weighing 48 kg, presented to the emergency department with complaints of pain, swelling, and limited joint movement in both elbows following a simple fall while playing. The fall occurred while running, during which his elbows suddenly flexed. Physical examination revealed blue sclera (Figure 1) and joint hypermobility.

The patient’s detailed medical history included multiple fractures treated conservatively: right tibia at seven months, right little finger at six years, right wrist at eight years, right clavicle at 10 years, and left tibia at 11 years. He had no other known health problems or comorbidities. Direct radiographic imaging of the elbows, wrists, shoulders, and clavicles revealed bilateral simultaneous displaced olecranon apophyseal fractures.

Management

**Figure 2:**
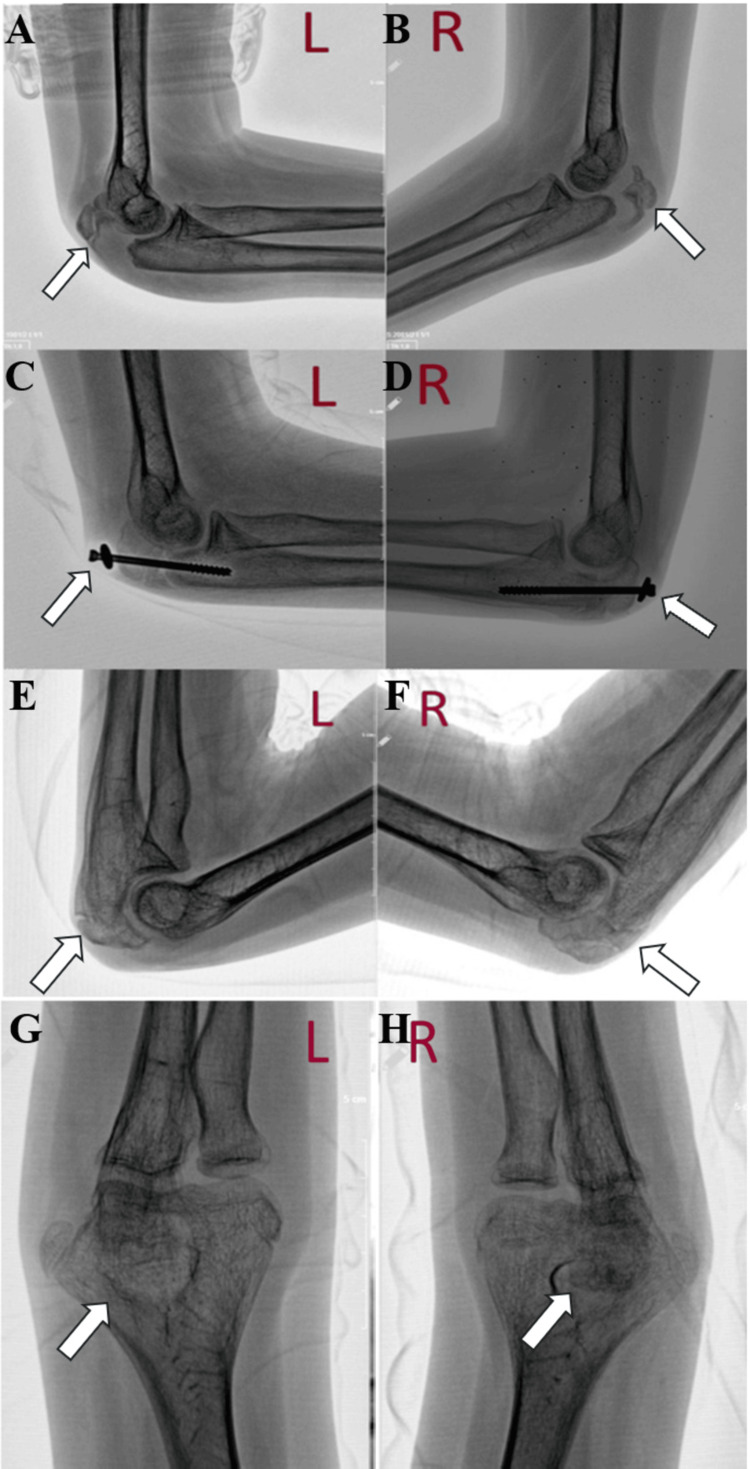
(A, B) Initial lateral plain radiographs of both the right and left elbows taken in the emergency room. (C, D) Lateral plain radiographs of both elbows at six weeks of follow-up. (E, F) Lateral radiographs of both elbows at one year of follow-up. (G, H) Both elbows' anteroposterior (AP) radiographs at one year of follow-up.

A successful operation was planned for the bilateral OFs. Under fluoroscopy and general anesthesia, both olecranons were operated on with a closed reduction and fixation using a cannulated screw and washer (Figure 2). Postoperatively, the patient was initially treated with a long arm splint (LAS) for two weeks, followed by two weeks with a sling. Passive range of motion exercises began after the LAS was terminated. Following the sling period, active-assisted exercises were introduced, and by the sixth week, active range of motion exercises commenced.

Clinical outcomes

**Figure 3:**
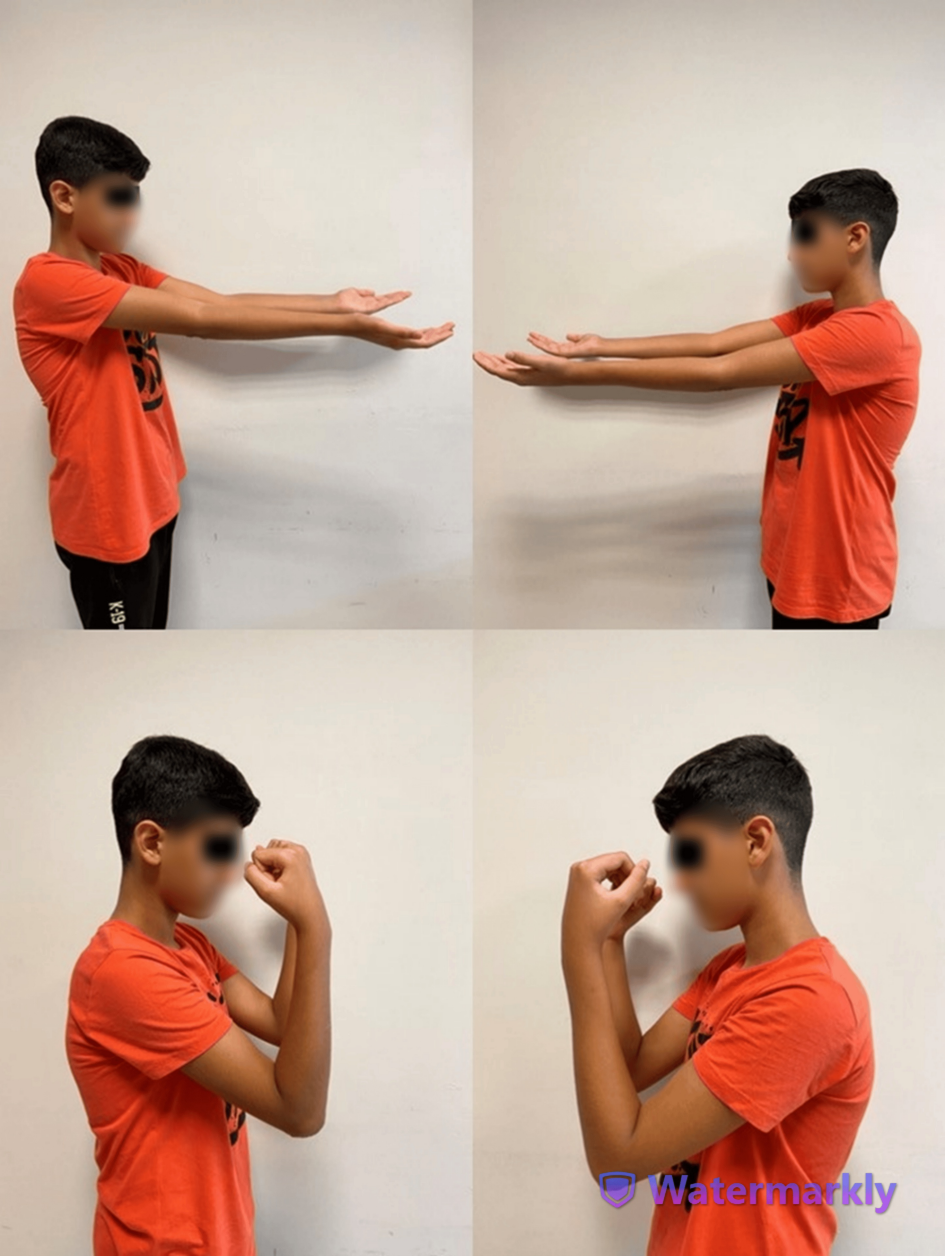
The patient's range of motion six months postoperatively.

During follow-ups, screw loosening was observed on both sides, which was more pronounced on the left side, but without loss of reduction. Continued follow-up resulted in the successful union of both fractures (Figure 2). Six months postoperatively, both elbow implants were removed under sedation analgesia and using fluoroscopy. The patient’s range of motion in both elbows was nearly complete and matched his pre-injury level (Figure 3). Genetic testing confirmed the presence of a COL1A1 mutation, thus confirming the diagnosis of OI.

## Discussion

OI, colloquially known as brittle bone disease, encompasses a range of hereditary disorders characterized by anomalies in collagen production metabolism. Type 1 OI is the most encountered clinically among the four primary types. Type 1 OI is characterized by mild bone fragility, blue sclera, and reduced collagen production. Mutations in the COL1A1 and COL1A2 genes, responsible for encoding type I collagen protein, contribute to 70%-80% of OI cases [6]. Despite type I collagen comprising 90% of the bone matrix, individuals with OI typically exhibit lower quality and quantity of this collagen. Within type 1, further distinctions exist, such as types 1A and 1B, differentiated by normal tooth development and dentinogenesis imperfecta, respectively [6,7].

Children with OI face an increased risk of fractures compared to the general population, with type 1 OI being the most prevalent subtype [3,8]. Decreased cortical bone thickness and increased vascular channels contribute to reduced diaphyseal strength in OI, predisposing individuals to fractures, particularly during adolescence when activity levels and growth rates are at their peak. However, fractures become less frequent in adulthood. The clinical variability of OI poses challenges in estimating its prevalence, as milder forms may go undetected or be diagnosed late [9,10].

In our case, although screw loosening occurred on both sides, there was no need for revision because reduction was maintained. However, due to the loosening, the implants were removed under sedation and analgesia in the same session at the six-month mark. The advantage of the screw technique includes the ability to perform closed reduction with minimal incision and to remove the implant through the same mini-incision. Conversely, the tension band wiring (TBW) technique is generally more challenging and requires a longer incision, but it has lower failure rates and rare instances of loosening. For these reasons, TBW may be the preferred choice [8].

The diagnostic significance of OF in OI remains a topic of debate. VanEenenaam et al. recently reviewed 403 pediatric patients with OF, finding only 14 (3.5%) with confirmed OI diagnoses, suggesting that isolated OF may not be pathognomonic for OI [11,12]. Conversely, other studies have noted that displaced isolated fractures of the olecranon apophysis are rare in healthy children, indicating a higher prevalence in children with OI, particularly type 1, with an incidence of 8.1% [3]. Moreover, children with type 1 OI who sustain an olecranon avulsion fracture are at an increased risk of contralateral involvement, with 5.7% experiencing bilateral asynchronous olecranon fractures [3,9].

Although unilateral and asynchronous bilateral olecranon fractures have been reported in the literature, synchronous bilateral olecranon fracture remains a rare occurrence [4,12]. Even though atypical fractures, such as olecranon fractures in childhood, may raise suspicion and necessitate investigation for OI, a definitive diagnosis for type 1 OI often takes considerable time. This delay can occur after several fractures have been conservatively treated, and in some cases, it may even lead to unnecessary referrals to child protection services. Recognizing the patterns and clinical signs associated with OI early on is essential to prevent such misunderstandings and to ensure timely and appropriate management for affected children [13].

This case report highlights the importance of clinical assessment alongside genetic testing for diagnosing OI. While a definitive diagnosis relies on genetic test results, a preliminary diagnosis can be strongly supported by basic medical evaluation, including history-taking, physical examination, recognition of disease-specific fracture patterns, hypermobility assessment, and sclera inspection. This approach is crucial for identifying OI patients who have not undergone genetic testing or for healthcare systems with limited resources for genetic testing.

Furthermore, recognizing the signs early on can serve as a crucial step before referring to child protective services, thereby preventing misunderstandings and unnecessary complaints [13]. Additionally, early suspicion and identification can prevent delayed diagnoses and poor management of type 1 OI, aiding in appropriate precautions and in formulating a comprehensive management plan tailored to the patient’s needs. This emphasizes the value of clinical acumen and thorough evaluation in diagnosing and managing conditions like osteogenesis imperfecta, even in settings where genetic testing may not be readily accessible.

## Conclusions

This case report is significant as it is, to our knowledge, the first documented instance in the literature of a patient being diagnosed with OI following bilateral simultaneous olecranon apophyseal fractures due to low-energy trauma. Although olecranon apophyseal fractures are not pathognomonic for OI, they should be included in the differential diagnosis portfolio of every orthopedic surgeon and serve as a warning sign. This rare case emphasizes the importance of taking a detailed medical history and adopting a holistic approach through a comprehensive physical examination.
